# USABILITY ASSESSMENT OF A SENSOR-CONTROLLED DIGITAL GAME FOR OLDER ADULTS WITH HEART FAILURE

**DOI:** 10.1093/geroni/igz038.3263

**Published:** 2019-11-08

**Authors:** Kavita Radhakrishnan, Christine Julien, Matthew O’Hair, Catherine Fournier, Grace Lee, Thomas Baranowski, Miyong T Kim

**Affiliations:** 1 School of Nursing, the University of Texas at Aust, Austin, Texas, United States; 2 The University of Texas Austin, Austin, Texas, United States; 3 Houston, TX, Houston, Texas, United States

## Abstract

The inability of older persons with heart failure (HF) to self-manage has contributed to poor health outcomes. Our team from nursing, digital game design, and mobile computing developed an innovative sensor-controlled digital game (SCDG) called ‘Heart Mountain’ to offer a portable, and enjoyable tool to facilitate engagement in HF self-management. We installed the SCDG application, which featured older adult game avatars on the participants’ smartphones. The SCDG utilized data from an activity tracker and weight scale to trigger game rewards, knowledge content and messages based on participants’ real-time behaviors. In this study we assessed the usability of a SCDG prototype with 10 HF older adults in Central Texas. Observations on the usability of the SCDG app by older adults were noted on a usability heuristics checklist. Acceptance and satisfaction were collected by an open-ended survey guided by Intrinsic Motivation Inventory after a week of playing the game. Participants (60% males, 60% white, ages 63-84) were able to play the game and use the devices after a training session that lasted for 15 minutes. We will present results on participants’ ease of use of the SCDG app, satisfaction with the knowledge content, quizzes and rewards features of the SCDG, and perceptions on acceptance and satisfaction with the SCDG for heart failure self-management. Our project will generate insights on designing digital gaming solutions that are acceptable to older adults and can be applied to improve self-management of chronic diseases like heart failure.

